# Gene Targeting Using Homologous Recombination in Embryonic Stem Cells: The Future for Behavior Genetics?

**DOI:** 10.3389/fgene.2016.00043

**Published:** 2016-04-11

**Authors:** Robert Gerlai

**Affiliations:** Department of Cell & Systems Biology and Department of Psychology, University of Toronto MississaugaMississauga, ON, Canada

**Keywords:** gene targeting, embryonic stem cell, homologous recombination, neuroscience, behavior genetics

## Abstract

Gene targeting with homologous recombination in embryonic stem cells created a revolution in the analysis of the function of genes in behavioral brain research. The technology allowed unprecedented precision with which one could manipulate genes and study the effect of this manipulation on the central nervous system. With gene targeting, the uncertainty inherent in psychopharmacology regarding whether a particular compound would act only through a specific target was removed. Thus, gene targeting became highly popular. However, with this popularity came the realization that like other methods, gene targeting also suffered from some technical and principal problems. For example, two decades ago, issues about compensatory changes and about genetic linkage were raised. Since then, the technology developed, and its utility has been better delineated. This review will discuss the pros and cons of the technique along with these advancements from the perspective of the neuroscientist user. It will also compare and contrast methods that may represent novel alternatives to the homologous recombination based gene targeting approach, including the TALEN and the CRISPR/Cas9 systems. The goal of the review is not to provide detailed recipes, but to attempt to present a short summary of these approaches a behavioral geneticist or neuroscientist may consider for the analysis of brain function and behavior.

## Introduction

A simple search in PubMed using the key words “gene targeting” and “mice” returns close to 30,000 entries (Jan 2016). This is a vast literature, a number that shows the popularity and the utility of this technology. Cross referencing the entries with the key word “brain”, reduces the number of hits to about 4000, still a large number of papers just within the field of neuroscience. By now, several thousand genes have been mutated using homologous recombination-based methods in embryonic stem (ES) cells (Capecchi, 2005), and companies as well as major Governmental funding agencies such as the National Institutes of Health of USA have made concerted efforts to generate and assemble a collection of such mutant mouse lines (Austin et al., 2004). Also, the International Mouse Knockout Consortium (IKMC) (http://www.knockoutmouse.org/) has amassed a large collection of conditional knockout alleles for mouse genes, allowing neuroscientists and other researchers to avoid having to go through the labor intensive process of generating null mutant mice. Undoubtedly, gene targeting with homologous recombination in ES cells has revolutionarized the analysis of gene function, and it had a major impact in biology that was recognized by awarding the Nobel Prize in Physiology or Medicine to the inventors who laid the foundation of the method, Mario R. Capecchi, Sir Martin J. Evans, and Oliver Smithies in 2007. The current review is not intended to capture the full impact of this powerful method. Instead, it summarizes some of the advantages as well as disadvantages of the methodology as they pertain to behavioral and brain research. The review provides a brief discussion of some of the principle and technical challenges that the technology faced in the past, the solutions that have been offered to address them, and the future of the technology in the light of new developments in the field of gene manipulation and genome engineering.

## Gene Targeting: Great Promise of Specificity

The late 1980’s witnessed the birth of an efficient method with which investigators could silence their gene of choice. The method was based upon homologous recombination between a targeting vector and the endogenous gene of interest (Smithies et al., 1985; Mansour et al., 1988; also see Capecchi, 2001). The efficiency of the method was due to two main factors. One, the selection for the appropriate gene targeting event was conducted in the Petri dish, using ES cells instead of whole organisms. Two, the selection included two main steps. The first step could identify those ES cells whose genome contained the incorporated targeting vector. This was achieved by engineering a neomycin resistance conferring cassette into the homology region of the targeting vector (usually in the region that would correspond to an important and upstream exon of the targeted gene) (Cheah and Behringer, 2000). The second step could identify those ES cells in which the incorporation of the targeting vector happened via homologous recombination, i.e., by replacing the endogenous gene, as opposed to insertion into a random locus (Cheah and Behringer, 2000). The latter step was achieved by inclusion of the thymidine kinase cassette usually downstream of the homology region of the construct (Mansour et al., 1988). This double selection scheme thus allowed the investigator to quickly and efficiently identify ES cells in which the gene of interest was replaced by the targeting vector. Importantly, because the targeting vector contained a non-native sequence, e.g., the neomycin cassette, in the middle of an important exon of the gene of interest, or had a stop codon upstream of coding regions, or both, when this targeting vector replaced the endogenous gene, there was either no protein expression from it, or the translated protein was structurally so abnormal that it could not serve the original biological function. Thus, the mutation induced with this technology was called null mutation, and the transgenic mouse carrying such a mutation, the knock out or null mutant mouse.

Knock out mice were an appealing tool for the neuroscientist, and with the first two mouse knock out studies published from the laboratories of two Nobel Laureates, Susumu Tonegawa (Silva et al., 1992), and Eric Kandel (Grant et al., 1992) in 1992, the technology gained foothold in neurobiology. The main appeal of these mice was that they possessed a genetically well defined change, a single silenced gene with all other biological targets (genes and gene products) intact. Or at least so was the thinking of that era. The principal reason why gene targeting was viewed as highly promising was that it offered an excellent alternative to pharmacological methods. The latter suffered from two fundamental problems. One, identification of compounds that would interact with biological systems or targets required large scale screening. The so called intelligent drug design, i.e., the ability to make custom designed small molecules that would specifically bind biological targets of interest in a desired manner, was not, and still is not, feasible. The second problem with pharmacological tools was that even after the binding affinity and efficacy of a particular small molecule have been confirmed, the specificity of this compound remained in question. After all, no one could tell for sure that a particular compound would not bind or interact with a yet undiscovered molecular target or biochemical pathway. Geneticists argued that for the above reasons, gene targeting is the way to go. If the nucleotide sequence of a gene was known, geneticists could custom design a targeting vector that would specifically and selectively disrupt the functioning of this, and only this target gene (Cheah and Behringer, 2000). But as it turned out, this argument was not entirely correct, at least not from the perspective of the main reason why gene targeting would be conducted: the analysis of the function of the gene, i.e., what role it may play in influencing the phenotype.

## Fundamental and Technical Issues of Past Gene Targeting Methods

There were two distinct problems with gene targeting, a technical, and a more fundamental scientific issue. I deal with the latter one first. The fundamental issue with gene targeting was what became known as the problem of compensation. Investigators occasionally noticed that despite clear and confirmed full silencing of their target gene, i.e., despite a lack of functional gene product, there was no observable phenotypical effect, as if the gene had no function at all (Müller, 1999). The reason for the latter, many argued, must have been compensation by “helper” genes, as it has been empirically shown (Hummler et al., 1994). The argument seemed reasonable given that we knew of many gene families within which genes (most likely generated by DNA sequence duplication events throughout evolution) would have sisters, genes with highly similar nucleotide sequences encoding proteins with highly similar, if not identical, functions. But the phenomenon of compensation brought up another, rather vexing issue. Some explained that the avalanche of compensatory changes induced by the absence of a gene product may manifest as secondary alterations at the phenotypical level that are not truly directly related to the actual function of the target gene (Gerlai, 1996b, 2000b). Let me illuminate the point with a hypothetical example. Consider a simple gene family with only two sister genes. Assume that these genes would express proteins with highly similar amino acid sequence, and thus protein function, but in a spatially slightly different expression pattern. A real life example would be the EphA-family tyrosine kinase receptors (Gerlai, 2001) (except that there are eight sister receptors in this family). These receptors are highly similar, and their spatial expression pattern is partially overlapping (Gerlai, 2001). Imagine we knocked out gene A1 and in response to this null mutation gene A2 gets overexpressed. In the brain region where both A1 and A2 would be expressed (the overlapping area, say, area X) gene A2 would thus be able to compensate for the absence of gene A1 product. However, note that the spatial expression pattern of sister genes is almost never completely overlapping. In the area where only gene A2 is expressed in the wild type animal (say area Y), now gene A2 may be overexpressed in the null mutant. This overexpression, may result in altered functioning of this brain area and this alteration may be observed at the level of behavior or any other phenotype. Thus, although the experimenter may properly conclude that knocking out gene A1 alters the functioning of brain area Y, the argument that the function of gene A1 is in area Y would be flawed.

The phenomenon of compensation, and the resulting secondary changes, is a vexing issue (Gerlai, 1996b). It undermines our ability to answer the question originally thought of as the main goal of gene targeting: what is the function of the gene in terms of its phenotypical effect. Compensation is a vexing issue also because there really is no appropriate solution for it. We can investigate the behavior of any system only when we interact with it. Thus, we can never know how the intact system would behave, i.e., how it was working before we interacted with it, a problem inherent in all research, not just biology. Although a complete solution to the above issue does not exist, one can still limit the effect of compensation and the ensuing secondary changes by restricting gene targeting temporally and/or spatially, and thus making the genetic manipulation more specific and controlled. This was achieved by the second generation gene targeting methods (Mayford et al., 1997), to which I will return later.

**FIGURE 1:**
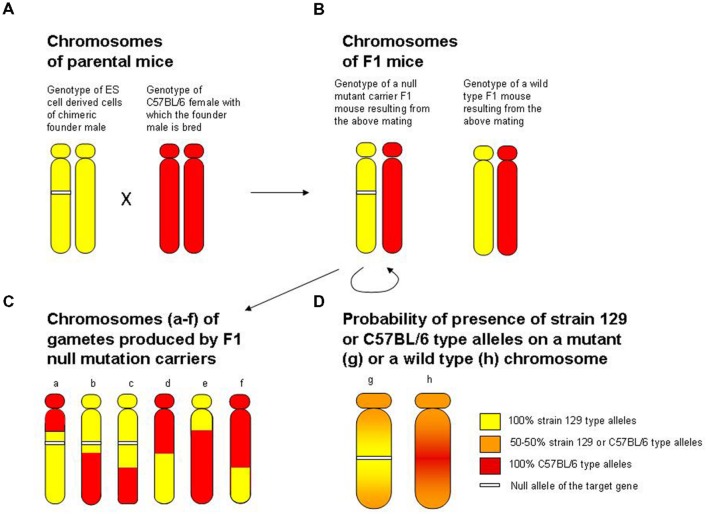
**The flanking allele (false positive finding) and the increased genetic variance (false negative finding) problems associated with the hybrid origin of null mutant mice**. For illustrative purposes, the figure focuses on the particular chromosome where the locus of the target gene is. **(A)** The genotype of diploid ES cell derived tissue of the chimeric founder male (yellow indicating strain 129-type alleles, white segment indicating the null allele at the target gene locus) and the genotype of the host female with which the chimeric male is mated (red indicating strain C57BL/6 type alleles). Note that if the testes of the male chimeric founder mouse have developed from the ES cell (germ-line transmitting chimera), this male will produce strain 129 genotype sperm in which the chromosome with the target gene locus will carry the null mutant allele with 50% probability. When a germ line transmitting male founder chrimera is crossed to a C57BL/6 female as indicated in **(A)**, individuals of the resulting F1 offspring generation **(B)** will be heterozygous for the strain 129-type versus strain C57BL/6 type alleles at all loci. Notably, 50% of these mice will also be heterozygous for the null allele, while the other 50% will not carry the null allele, and will have one wild type allele from the strain 129 genome and one wild type allele from the C57BL/6 genome at the locus of the target gene. **(C)** Shows the genotype of gametes (sperm or egg) of the F1 mice. During meiosis, recombination will shuﬄe the strain 129-type and C57BL/6 type alleles, resulting in recombinant chromosomes indicated by the yellow (strain 129-type) and red (strain C57BL/6-type) segments. In order to obtain homozygous null mutant mice, two F1 heterozygous null mutant individuals are mated (indicated by the semicircle underneath the heterozygous null mutant F1). The genotype of F2 individuals (offspring from this F1 sibmating) thus can be described as pairs of any chromosomes illustrated on **(C)**. Note that the random recombination pattern induced genetic variance in this F2 segregating generation may make the effect of the null mutation less detectable (leading to a false negative finding). Also note that chromosomes that carry the null allele (chromosomes a, b and c with the white segment) have a strain 129-type region (yellow) surrounding the target gene locus, and that chromosomes that do not carry the null allele (chromosomes d, e and f) have a C57BL/6-type region (red) surrounding the target gene locus. This pattern of recombination is simply due to that recombination occurs essentially randomly, and to that the shorter the length of DNA the less likely that recombination occurs within that area. Thus, the closer one gets to the target locus that carries the null mutation, the less likely a recombination event will be to occur, a phenomenon known in classical genetics as linkage. Therefore, because the homologous recombination-based gene replacement event was achieved in the strain 129-type genome of the ES cell, the null allele will be surrounded by strain 129-type alleles, whereas the wild type allele of the target locus (which originates from the C57BL/6 genome) will be surrounded by C57BL/6 type alleles. **(D)** Summarizes this, and illustrates that the closer one gets to the target locus carrying the null allele (chromosome g) the more likely one will find strain 129-type alleles, whereas the closer one gets to the target locus carrying the wild type allele (chromosome h) the higher the probability will be for one to find C57BL/6 type alleles. This problem has become known as the flanking allele or hitch-hiking gene issue. The flanking allele issue is problematic because the null mutant mice and their wild type siblings differ in two ways: first, the former carries the null mutation while the latter does not; and second, the former has strain 129-type alleles around the target locus while the latter has C57BL/6 type alleles around the target locus. Thus, one may falsely conclude that the phenotypical difference between null mutant and wild type mice is due to the null mutation, when, in fact, it is the result of differences in alleles of genes whose loci reside in the, often large, flanking region surrounding the target locus. Adapted from Gerlai (1996a).

The second major problem with gene targeting, which was pointed out already 20 years ago, concerns the hybrid origin of the knock out mice (Gerlai, 1996a). This problem became known in the literature as the flanking allele or hitchhiking gene issue. The issue is a simple classical genetic phenomenon, linkage, but one that was largely missed or ignored, and is still often ignored in gene targeting studies (**Figure 1**). At the heart of the problem lies our limitation of what type of ES cells may be available for gene targeting. Briefly, the problem is as follows. Most ES cells that have been developed for gene targeting purposes come from substrains of mice called 129 (Nagy et al., 1993; Simpson et al., 1997; Downing and Battey, 2004). It is not entirely clear why strain 129 mice would allow the generation of ES cells that are appropriate for the purposes of gene targeting (it may be due to purely historical reasons, or perhaps to the unique genetic make up of these strains). But the fact remains that most currently available, and previously used, ES cells do come from strain 129 mice (Papaioannou and Johnson, 1993). The problem with this strain origin, especially for behavior or brain researchers, however, is that strain 129 mice exhibit peculiar phenotypical features. For example, these mice are extremely passive, they do not perform well in several behavioral tasks including certain learning paradigms (Crawley et al., 1997), and they have numerous peculiar abnormalities in their brain, including significantly diminished or completely absent corpus callosum (Bohlen et al., 2012). For these reasons, behavioral and brain scientists preferred using mice of other strains in their studies. But because the ES cell in which the homologous recombination event replacing the target gene occurred was from the strain 129 mouse, they were forced to work with this phenotypically abnormal animal. The simple solution seemed to be to cross the ES cell derived germ line transmitting Chimera (the male mouse in which the ES cell carrying the null mutation gave rise to the testes and thus the sperm) to a female mouse of the preferred strain origin, most often the C57BL/6 mouse to create an F1 hybrid. While this cross did solve the phenotypical issues, as the F1 hybrid did not exhibit the strain 129 specific peculiarities, it brought about two main problems, which I will discuss shortly. But first consider that the null mutation rarely manifests in a heterozygous form, because the presence of the null allele is usually fully compensated for by the presence of the wild type allele on the sister chromosome in the F1 hybrid. Thus, in order to see the effect of the null mutation, it often had to be in a homozygous form. To achieve this, an F1 male and an F1 female had to be mated to generate the F2 generation, in which 25% of the offspring carried the null mutation in the homozygous form. This breeding scheme (strain 129 ES cell carrying the mutation giving rise to the germ line transmitting chimera, chimera crossed with C57BL/female, F1 hybrid offspring mated with each other generating the F2) became the gold standard of making stable homozygous null mutant mouse lines. This breeding scheme generated two main problems, one that led to false negative and the other to false positive findings.

False negative findings arose because the F2 generation was a genetically segregating generation in which genetic variance was increased compared to the original parental strains of the F1 generation. This was due to the fact that individuals of this generation carried genetic recombinant chromosomes, mixture of genes from two distinct strains, the strain 129 type and the C57BL/6 strains. The problem with increased genetic variance is that it makes detecting null mutation effects more difficult. That is, compared to within population variance, the difference between null and wild type mice may be relatively small, leading to reduced statistical power to find the difference, hence the false negative finding.

The false positive finding aspect of the hybrid background, however, is even more troublesome. It results from the fact that the homologous recombination based gene replacement event occurred on the strain 129 genetic background. When strain 129 × C57BL/6 F1 hybrids are mated with each other, during the meiotic cell division generating the gametes of these mice, recombinant chromosomes are produced. According to the basic principles of genetic recombination, the probability of a recombination event to occur in a region of DNA is proportional to the length of this DNA region. This means that the closer we get to the targeted locus carrying the null allele, the lower the probability of recombination and thus higher the chance that we will find strain 129 type alleles, a phenomenon known in classical genetics as linkage. Similarly, the closer we get to the targeted locus carrying the wild type allele, the higher the chance that we will find C57BL/6 alleles. Briefly, due to genetic linkage the null allele may be viewed as a marker for the strain 129 region and the wild type allele of that locus may be viewed as a marker for C57BL/6 region of the given chromosome.

What does this all mean in terms of the analysis of null mutant mice, and why is this linkage issue a problem? It is a problem for gene targeting studies because the homozygous null mutant mice and the homozygous wild type control counterparts (littermates in the very same F2 segregating generation) differ in two ways. One, the null mutant mice carry the null allele and the wild type mice do not, and two, the null mutant mice have strain 129 type alleles around the target locus, but the wild type mice have C57BL/6 alleles around the target locus. Briefly, the scientist who is comparing null mutant and wild type mice can never decide whether the phenotypical difference between these two groups of animals is the result of the null mutation or of the genetic difference in the regions flanking the target locus (Gerlai, 1996a). This ambiguity may not represent a fundamental problem for phenotypes where major gene effects leading to robust changes are studied. However, for behavioral characteristics, including mnemonic and cognitive features, emotional or personality traits, and for many other neuroscience and also other subtle biology related phenotypes expected or known to be influenced by a large number of genes, the flanking allele problem questioned the validity of the interpretation of gene targeting results, and thus became a major issue in neuroscience studies (Gerlai, 1996a, 2000a). Notably, since the theoretical possibility of the flanking allele issue was raised in 1996, empirical evidence has been obtained multiple times that suggests the issue is real. For example, most recently Vanden Berghe et al. (2015) exemplified the phenotypic effects of 129-derived passenger mutations with several case studies, and these authors also developed a web tool to estimate the number and possible effect of the hitch-hiking genes in transgenic mice. Others also demonstrated background genotype dependent null mutation effects (Kõks et al., 2009) and experimentally showed the importance of the flanking region problem in null mutant mice, the “congenic footprint”, even after several backcross generations (Schalkwyk et al., 2007).

One may argue that second generation gene targeting, i.e., approaches in which inducible or cell type restricted gene targeting is achieved, provides proper answer to the flanking allele issue. For example, the ability to temporally control gene expression in transgenic mice with the use of the tetracycline transactivator or the reverse tetracycline transactivator systems (Mansuy et al., 1998), allows one to conduct a within subject comparison, i.e., comparison of “before and after” mutation phenotypes of the particular subject. Similarly, restricting the genetic manipulation to particular tissues, e.g., to particular brain regions using the CRE-recombinase/LoxP system (e.g., Tsien, 2016) or a conceptually identical Flp recombinase/FRT system (Andrews et al., 1985; Dymecki, 1996) would allow one to compare null mutant mice in which different brain areas are affected. Thus, one could argue that the flanking allele effects in these null mutant mice should be the same, and any differences among or between them must be the result of the differential brain region dependent null mutation effects. The problem with all of these arguments, however, is that they assume the effects of the null mutation and of the flanking alleles are additive. However, the assumption of lack of interaction between such genetic effects is almost certainly wrong.

And this is where we were about 15–20 years ago. What has changed? Have gene targeting studies altered their methods to address the above? How can the above problems be addressed anyway? I will briefly discuss these questions in the following pages, and subsequently will evaluate the homologous recombination in ES cell-based gene targeting technology in the light of more recent alternative methods.

## Solutions for the Flanking Allele Problem

Since the gene targeting debate in 1996 (Crawley, 1996; Crusio, 1996; Gerlai, 1996a,b; Lathe, 1996), several solutions have been offered for the flanking allele problem. In fact, an entire conference was organized to discuss and address this problem (Banbury Conference, 1997). However, admittedly, most of the recommendations were able to address the issues only partially. The simplest, and most often employed, “solution” is to backcross the heterozygous null mutant to the preferred strain, most often the C57BL/6 mouse. Backcrossing for 10 generations, for example (well over a year long effort) would eliminate most of the strain 129 alleles making the resulting backcross animal’s genome 99% C57BL/6 type. Even better, the elimination of strain 129 type alleles may be accelerated if one tests for genetic markers and selects the most C57BL/6 like individuals for breeding at every generation of the backcross, a technique that became known as speed congenics (Wong, 2002). The problem with this approach, however, is that the closer one gets to the target locus, i.e., the shorter the DNA region is around the targeted gene, the more difficult (less probable) it will be to find a recombination event, i.e., to exchange strain 129-type alleles with C57BL/6 type alleles via random recombination. Briefly, to fully eliminate the possible contribution of strain 129-type flanking alleles to the phenotype of null mutant mice, one would need to backcross the mutants for infinite number of times. Despite these limitations, numerous gene targeting papers employed the backcross and glanced over the possibility that the phenotype of the null mutant mouse may still be influenced by 100s or perhaps thousands of flanking genes around the target locus, a problem that continues to haunt gene targeting studies even today.

Another solution, offered by Zimmer (1996), was to create a control mouse population, using a breeding scheme identical to that used for the generation of the null mutant mice. For example, in addition to sibmating F1 heterozygous null mutants, in this method one would also sibmate two wild type F1 individuals. Since both the heterozygous null mutant and the wild type F1 mouse has one set of chromosomes from strain 129 and the other from the C57BL/6 strain, the two F2 populations would, on average, contain the same recombinant genetic patterns, i.e., the same genetic make up. The problem with this argument, however, is again genetic linkage. By choosing the homozygous null mutant mice from the F2 generation resulting from the F1 heterozygous matings one is essentially selecting mice whose target locus is surrounded by strain 129-type alleles. Whereas breeding wild type F1 mice that carry no null mutation will result in F2 generation mice whose targeted locus is not surrounded by strain 129-type alleles more than at random chance, which leads to a similar flanking allele issue to when one compares the wild type and null mutant mice form the same segregating F2 generation.

The third solution offered was what we call a rescue experiment. If one replaces the missing protein, via systemic delivery, or via adding a normally functioning transgene producing the endogenous gene product, and if this manipulation reverses (rescues) the phenotypical changes seen in the null mutant mice, one has proven that the phenotypical changes were indeed due to the null mutation. However, this extra (and quite labor intensive) step, i.e., the rescue experiment, is almost never attempted because the failure rate is expected to be quite high especially compared to the efforts required.

The second generation gene targeting methods, i.e., those that utilize temporally controllable and/or cell type restricted silencing of target genes (see e.g., current special topic paper on the CRE recombinase system by Tsien, 2016) have gained increasing use in behavior and brain studies. These approaches do offer the ability to create internal controls as explained above, and thus were argued to address both the flanking allele and the compensatory responses problems (Mayford et al., 1997). The issue with many of these studies, however, again relates to the hybrid origin of the mice used. For example, in case of the Cre-lox system, one needs to generate a transgenic line in which the Cre recombinase encoding transgene is driven by a desired promoter, for example, the CamKII promoter, which directs expression mainly to the forebrain and largely after birth of the mouse. This transgenic animal must be crossed to another transgenic mouse, in which the endogenous gene has been replaced (using homologous recombination in ES cells) by a construct that contains the entire target gene flanked by lox p site sequences, the recognition sequences for the Cre recombinase. Once this “floxed” gene is in the same genome as the CamKII-Cre construct, the Cre will excise the floxed target gene, resulting in complete silencing of the target gene only in tissues where and when the CamKII promoter directed the expression of Cre (hence the cell type specificity). This elegant technique, which has gained widespread use (see current review in this special topic by Tsien, 2016), however, may still suffer from the same, or perhaps even more complicated genetic background (hybrid origin) problem as null mutant mice generated by classical, first generation, gene targeting. This is because the line containing the floxed gene is generated the same way as standard knock out mice, and thus has the strain 129 vs. C57BL/6 background issue, and two, because the transgenic line carrying the Cre construct is often of yet another strain origin.

Perhaps the only true solution to the flanking allele and also to the false negative problems is to completely eliminate the hybrid origin of the knock out mouse. By using an ES cell line that originates from the same mouse strain to which the germ-line transmitting mutant chimera is crossed, one generates null mutant mice that are on a pure bred, genetically homogeneous, background. Although relatively rare, ES cells of strains other than 129 substrains have been successfully generated. For example, ES cells have been developed from inbred mouse strains most often utilized in neurobehavioral genetic research, which include C57BL/6 ES cell lines (Tanimoto et al., 2008), BALB/c ES cells (NobenTrauth et al., 1996), and DBA strain derived ES cells (Roach et al., 1995). These alternative ES cells often require different maintenance protocols and/or may not populate the founder chimera as efficiently as strain 129 derived ES cells (Ledermann, 2000), but they save all the ambiguities associated with the traditional ES cell lines.

Last, another major limitation of the above discussed gene targeting methods is that they are species specific. They have been developed for the house mouse and cannot be easily adopted for other species. The main limitation is the lack of availability of ES cells, which are crucial for the efficient screening of the appropriate gene targeting event. Recently, however, ES cells have been developed for the rat, and thus transgenic and null mutant rats have started to be utilized in brain research (Kawaharada et al., 2015). This is a major step for biomedical neuroscience research, because most traditional psychopharmacological and toxicology studies have been conducted with this latter species. Briefly, we know much more about the physiology, behavior and neurobiology of the rat as compared to what we know about the house mouse.

## Will Homologous Recombination-Based Gene Targeting Survive the Test of Times?

The huge number of publications in which homologous recombination in ES cells based gene targeting is utilized clearly attests to the great utility and popularity of this technique. Thus, perhaps the above critical comments may be too harsh. Increasingly sophisticated ways of targeting genes in an inducible (Mishina and Sakimura, 2007) and cell type restricted manner (Mayford et al., 1997) also suggest that the method has a future. New developments in the methods of how one can generate genetically and/or epigenetically reprogrammed pluripotent cells (Theunissen and Jaenisch, 2014) that can function as ES cells will likely open the possibility of using gene targeting in numerous species in addition to the traditional laboratory rodents. Even the classical double selection method has been modified. For example, an elegant auto-selection targeting vector has been designed (Bouabe et al., 2011) in which the CRE recombinase encoding sequence driven by a herpes simplex virus (HSV) promoter was positioned outside the homology region of the targeting vector, and the neomycin resistance gene cassette was engineered with two loxP sites flanking it (floxed neo). In case of random integration of this targeting vector, the CRE sequence is expected to be incorporated into the genome. The resulting expression of CRE recombinase excises the floxed neo and thus such ES cells die in response to antibiotic treatment. However, in case of homologous recombination, the CRE sequence is expected to be lost, and the intact incorporated neo sequence is expected to confer antibiotic resistance to such ES cells, hence the ES cells are easily selectable for the homologous recombination event with a single selection step, antibiotic application. Last, but also notably, inducible gene expression systems and cell type restricted conditional knock out may be combined to achieve inducible AND cell type restricted conditional null mutation. This system is essentially a CRE/loxP system in which CRE expression is rendered inducible, i.e., temporally controllable. For example, one can fuse a nucleotide sequence encoding the mutated ligand-binding domain of the estrogen receptor (ER) with the CRE sequence. Tamoxifen specifically binds this mutated ligand-binding domain of the ER. In response to tamoxifen treatment, the CRE-ER fusion protein translocates from the cytoplasm to the nucleus, where it can excise the loxP site flanking target gene (Feil et al., 1996). A conceptually similar idea was to combine the tetracycline transactivator system with the CRE recombinase system. In this method, CRE expression is driven by a minimal promoter with the tetracycline transactivator operator, TetO, which may turn on CRE expression when tetracycline or similar antibiotic analogs (e.g., doxicycline) are removed from the diet or drinking water of the transgenic mouse (Shimizu et al., 2000; Lindeberg et al., 2002).

Given such advances in gene targeting, the method originally developed by the three pioneering Nobel Laureates, Capecchi, Smithies and Evans, will likely withstand the test of times. Nevertheless, it is also likely that in the future new recombinant DNA methods, including the TALEN (transcription activator-like effector nuclease) and the Clustered regularly interspaced short palindromic repeats (CRISPR)/Cas9 (clustered regularly interspaced short palindromic repeats) gene targeting systems (Boettcher and McManus, 2015; Pu et al., 2015; also see current special topic paper by Walters et al., 2016), will gain increasingly strong roles in the genetic analysis of brain function and behavior. One reason why these newly developed techniques may become more frequently used at the expense of classical gene targeting is that even though true-and-tried, the ES cell-based gene targeting method is more time consuming, labor intensive and expensive than many of the recently developed genome editing tools.

In addition, although not considered a gene targeting method *per se*, optogenetics (which allow light controlled activation or deactivation of particular neuronal circuits, Gross (2011)) and promising combinations between pharmacology and genetics, such as DREADDS (which utilizes genetically modified channels or receptors designed to bind particular artificial ligands, Whissell et al. (2016)), may also enrich the toolset of the neurobiologist leading to unprecedented sophistication with which the central nervous system may be probed. Some of these techniques are discussed in this very issue/special topic (Lee et al., 2016; Walters et al., 2016; Whissell et al., 2016), others have been reviewed and explained in detail elsewhere. Thus, I only briefly discuss the novel gene targeting systems, particularly in the context of how they compare to classical gene targeting.

## Alternatives of Homologous Recombination in ES Cell-Based Gene Targeting

Reverse genetics with the TALEN system has one major advantage over homologous recombination in ES cell based gene targeting. It can be conducted in any species. For example, while classical gene targeting is currently restricted to the house mouse and recently extended to the rat, the TALEN-based gene targeting method is not dependent upon the availability of ES cells, and has already been successfully utilized in a range of species, including, for example, one of the simplest vertebrates, the zebrafish (Clark et al., 2011; Lee et al., 2016). The method utilizes the transcription activator-like effector proteins or TALEs, which are site-specific DNA-binding proteins discovered in Xanthomonas species, bacterial pathogens of plants. TALEs are simple proteins consisting of 34 amino acids that recognize a specific DNA base. Particular nucleotide sequence recognition is achieved by building a series of TALEs (TALE repeats) specific to the target sequence. Cutting of DNA at the specific target sequence is accomplished by fusion of the catalytic domain of the FokI endonuclease to the TALE repeats, a protein called transcription activator-like effector nuclease or TALEN. Because the FokI endonuclease requires dimerization in order for it to induce double stranded cutting of DNA, two TALENs are designed, one to recognize a nucleotide sequence on one side, say, 5′ to the cut site, and another to recognize nucleotide sequence 5′ to the cut side on the other strand of the DNA, with about a 15 base pair gap in between these recognition sequences in the middle. Due to DNA repair mechanisms, the double stranded cut is quickly joined, but the repair is error prone often resulting in insertion or deletion of nucleotides at the cutting site leading to frame shift mutation, i.e., altered reading frame, which renders the protein translated from this mutated gene functionally null. The TALEN technology can also be used to insert short nucleotide sequences at the cutting site using sequences that have a few hundred base-pair long overlapping homologous sequences on each side of the cut. Insertion of such sequences may be employed to correct a mutant gene, to insert a sequence mimicking a specific human mutation, and/or to insert reporter sequences, for example encoding the green fluorescent protein, in addition to disrupting a target gene. The latter not only allows efficient generation of null mutations, but when the insertion is targeted immediately downstream of the promoter of the gene of interest, it allows one to map the expression pattern of the target gene.

Although fundamentally a reverse genetic technique, the TALEN method may be utilized as a forward genetic approach too. Given the relative ease with which a large number of genes may be mutated, one can quickly generate a library of mutants and screen for interesting mutation effects identifying genes involved in particular functions. Furthermore, when combined with the use of reporter constructs, one can focus the phenotypical characterization to the most relevant mutants. For example, in case of brain or behavioral research, the investigator may ignore those mutants in which GFP expression driven by the promoter of the mutated target gene is found in organs other than the brain, a strategy successfully utilized in zebrafish, for example (Clark et al., 2011). In summary, the TALEN technology is a new, but rapidly evolving, genome engineering tool, which may be employed in a flexible manner serving a variety of different purposes in reverse as well as forward genetic studies conducted with species not accessible for homologous recombination in ES cell based gene targeting methods.

Nevertheless, as it employed now, the TALEN system has a potential minor disadvantage over homologous recombination based gene targeting: specificity. The number of TALE repeats employed is usually not higher than 24, and thus the length of recognition nucleotide sequence is quite short (also not more than 24 nucleotides). Thus, the possibility of off-target effects cannot be completely excluded. This is not a problem in homologous recombination based methods where the homology region of the targeting vector is much longer, as it often spans most, if not all, of the target gene. In fact, the importance of nucleotide sequence homology is well illustrated by findings showing that the most successful homologous recombination is achieved when the targeting vector is generated from a genetic background that is isogenic with the ES cell’s genome in which the homologous recombination based gene replacement is supposed to occur (te Riele et al., 1992).

The currently available CRISPR systems suffer more from the problem of potential off-target effects compared to the TALEN system, because the nucleotide sequence conferring specificity is even shorter (not more than 20 nucleotides). Nevertheless, the advantage of the CRISPR system over the TALEN method is that it is comparably simpler to perform, and thus its use is rapidly spreading across molecular biology laboratories. Furthermore, similarly to the TALEN system, it allows genome engineering in a variety of species. The CRISPR/Cas9 system was developed for genome engineering after the discovery of a defense mechanism that naturally occurs in bacteria (Doudna and Charpentier, 2014). CRISPRs have been discovered in the bacterial genome, and in between these short nucleotide sequence repeats viral or plasmid DNA may integrate from pathogens invading the bacterium (Fineran and Charpentier, 2012). The integrated foreign nucleotide sequence provides a template for the transcription of a hybrid RNA molecule (crRNA) that spans the incorporated foreign sequence from the invading pathogen and sequences from the CRISPR repeats flanking the foreign sequence. Following transcription, crRNA hybridizes with a second RNA (the transactivating CRISPR RNA or tracrRNA) and forms a complex with the Cas9 (CRISPR associated protein 9) nuclease. Unlike restriction endonucleases, Cas9 has no native preference for cutting at particular nucleotide sequences. Its cutting specificity comes from the incorporated foreign sequence flanked by the CRISPRs of the crRNA, which allows Cas9 to create a double stranded break specifically at the sequence of the pathogen’s DNA corresponding to the incorporated sequence. Once this mechanism was understood, it was quickly realized that this natural defense system may be utilized for creating double stranded breaks at a sequence of choice. In the CRISPR/Cas9 gene targeting system, the crRNA and tracrRNA is fused into a single construct and this guide RNA, or gRNA complexes with the Cas9 endonuclease that now targets the specific sequence engineered into the crRNA portion of the gRNA. The end result is a double stranded break specifically at the 20 nucleotide long sequence of choice. In other words, the cutting specificity of the Cas9 endonuclease has been made programmable (Doudna and Charpentier, 2014). Non-homologous end-joining of DNA at the double stranded break is highly error prone and often leads to insertion or deletion of nucleotides leading to a shift in the reading frame, which is expected to have a major disruptive effect on the structure and thus the function of the protein translated from this mutant gene. In addition, if a donor DNA is added which has sequences that match those that flank the site of the double stranded break, homology-directed repair will lead to the incorporation of the donor DNA. The latter thus allows the delivery of short nucleotide sequences at a locus of choice, which, similarly to what may be achieved with the TALEN technology, may allow one to introduce specific mutations (e.g., mutation from a human gene) or to repair mutations of the gene of interest. The reason why the CRISPR/Cas9 technology is relatively simple compared to other genome editing tools developed is that the cutting specificity conferring nucleotide sequence (the gRNA) is easy to design, and irrespective of its specific sequence it always uses the same Cas9 endonuclease. Last, it is also notable that recently, point mutated designer versions of Cas9 have been created that are reported to increase specificity in cell culture (Kleinstiver et al., 2016; Slaymaker et al., 2016). If these pioneering efforts are successfully adapted to genetically engineered animals, the enhanced Cas9 variants along with improved software for designing sgRNAs may significantly reduce the possibility of off-target effects associated with this emerging technology.

Are these rapidly evolving novel genome engineering methods always superior to homologous recombination in ES cells-based gene targeting? Not necessarily. The latter still has utility for the generation of conditional, cell type and temporally controlled null mutations. Furthermore, if the human gene is complex, large, and has multiple mutations, homologous recombination in ES cells based gene targeting is still a method of choice as it allows the knock in of the entire human gene, i.e., the generation of the genetic disease model in mice or rats. However, the faster and more efficient reverse and forward genetic methods including the TALEN and CRISPR/Cas9 systems will likely continue to gain increasing sophistication and role in the analysis of brain function and behavior and soon may take the lead in the laboratory of molecular biologists.

In summary, the molecular neurobiologist of today has a great selection of gene targeting methods available. Second generation inducible and cell type restricted gene targeting in ES cells continues to increase in terms of methodological sophistication and thus remains one of the best techniques for probing the brain. Nevertheless, new genome engineering methods including the CRISPR/Cas9 and TALEN systems will likely play increasingly important roles in the analysis of brain function and behavior. These latter methods will not only make gene targeting relatively easier and simpler to achieve, but they will also extend genetic manipulation of brain function to a range of species other than the traditional rodent laboratory research organisms.

## Author Contributions

The author confirms being the sole contributor of this work and approved it for publication.

## Conflict of Interest Statement

The author declares that the research was conducted in the absence of any commercial or financial relationships that could be construed as a potential conflict of interest.
